# Automatic Detection of Fiducial Landmarks Toward the Development of an Application for Digitizing the Locations of EEG Electrodes: Occipital Structure Sensor-Based Work

**DOI:** 10.3389/fnins.2021.526257

**Published:** 2021-04-29

**Authors:** Elieser E. Gallego Martínez, Anisleidy González Mitjans, Eduardo Garea-Llano, Maria L. Bringas-Vega, Pedro A. Valdes-Sosa

**Affiliations:** ^1^The Clinical Hospital of Chengdu Brain Sciences, University of Electronic Science and Technology of China, Chengdu, China; ^2^Telecommunications and Electronic Department, University of Pinar del Río “Hermanos Saíz”, Pinar del Río, Cuba; ^3^Mathematic Department, University of Havana, Havana, Cuba; ^4^Cuban Neuroscience Center, La Habana, Cuba

**Keywords:** Index Terms-Anatomical landmarks, automatic fiducials detection, computer vision, structured sensor, EEG sensor coordinates

## Abstract

The reconstruction of electrophysiological sources within the brain is sensitive to the constructed head model, which depends on the positioning accuracy of anatomical landmarks known as fiducials. In this work, we propose an algorithm for the automatic detection of fiducial landmarks of EEG electrodes on the 3D human head model. Our proposal combines a dimensional reduction approach with a perspective projection from 3D to 2D object space; the eye and ear automatic detection in a 2D face image by two cascades of classifiers and geometric transformations to obtain 3D spatial coordinates of the landmarks and to generate the head coordinate system, This is accomplished by considering the characteristics of the scanner information. Capturing the 3D model of the head is done with Occipital Inc. ST01 structure sensor and the implementation of our algorithm was carried out on MATLAB R2018b using the Computer Vision Toolbox and the FieldTrip Toolbox. The experimental results were aimed at recursively exploring the efficacy of the facial feature detectors as a function of the projection angle; they show that robust results are obtained in terms of false acceptance rate. Our proposal is an initial step of an approach for the automatic digitization of electrode locations. The experimental results demonstrate that the proposed method detects anatomical facial landmarks automatically, accurately, and rapidly.

## Introduction

An important goal for electroencephalography (EEG)-based functional brain studies is to estimate the locations of brain sources produced by scalp-recorded signals. The localization of such sources requires precise knowledge of the EEG sensors coordinates, while the employed method should be accurate, fast, reproducible, and cheap (Koessler et al., 2007).

**FIGURE 1:**
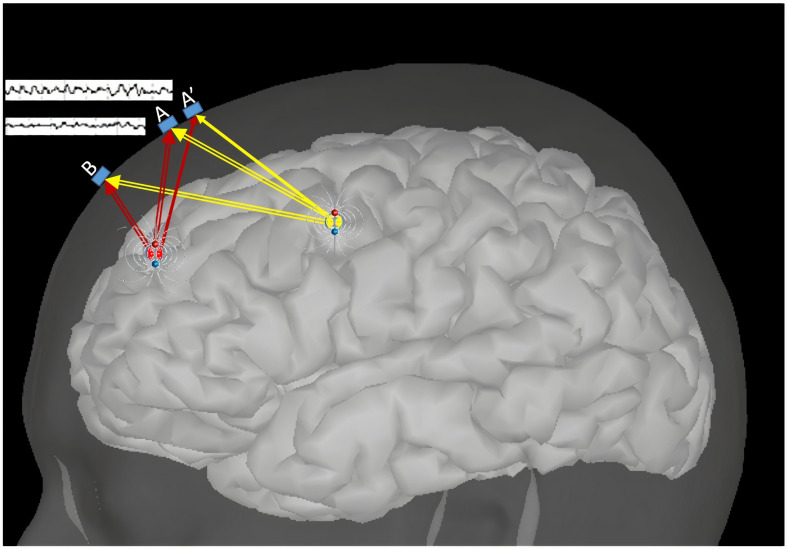
Effects of sources on EEG sensors, the dependence on the electric field overlay, and the electrode positions.

The coordinates of EEG electrodes are highly relevant to the solution of the inverse problem. Inaccurate sensor co-registration (and, by extension, a poor head model quality) not only will result in the mislocalization of source locations but also may prevent the detection of relatively weak signals entirely (Dalal et al., 2014); this may be the case even when the determined positions are only slightly different from the correct ones. Figure 1 compares examples of incorrect and correct position vectors (A’ and A, respectively). Nevertheless, the required level of accuracy for EEG electrode localization is still unclear (Koessler et al., 2007).

There are several techniques to digitize the electrode locations; among them, some manual methods are used, such as

• Direct measurement (De Munck et al., 1991) determines the position between each sensor and fixed landmarks (nasion and left and right preauricular points) with a caliper (Binnie et al., 1982; Koessler et al., 2007).
• Measuring interelectrode distances; these measurements are also performed using calipers. This technique assumes that the EEG electrodes are positioned in a defined configuration corresponding to the 10–20 or 10–10 International System (Klem, 1961; Chatrian et al., 1985; Oostenveld and Praamstra, 2001).
• Free placement of electrodes. A free electrode placement tool that is available in the FieldTrip Toolbox; a MATLAB-based script (Oostenveld et al., 2011). This facility has been added to the EEG-LAB toolbox as a new plugin (Delorme and Makeig, 2004). This particular technique was incorporated into our work for some operations, as we will mention later.

An alternative to the aforementioned manual techniques is known as electromagnetic digitization, based, for example, on the Fastrack system (US Patent “Magnetic Sensor System For Fast Response, High Resolution, High Accuracy, Three-Dimensional Position Measurements”) (Nelson and Jacobs, 2004). The Fastrack system constitutes a 3D procedure that uses a magnetic field to localize EEG electrodes. The system has a transmitter device that produces the electromagnetic field and simultaneously incorporates a geographical reference for the positioning and orientation of the receivers. In this system, three receivers are placed on the head to conduct measurements (Koessler et al., 2007, 2010; Engels et al., 2010).

All the alternatives described previously have one aspect in common: they need to be operated manually, which can be considered time consuming and may imply a remarkably high economic cost.

In this work, we present a method for automatic estimation of fiducial landmarks for the automatic location of EEG electrodes on a 3D human head model captured by a standard commercial sensor. Our proposal combines dimensionality reduction with a perspective projection from 3D to 2D object space; the detection of anatomical face elements (eyes, mouth, ears) in a 2D face image by machine learning classifiers and geometric transformations to obtain 3D spatial coordinates of the landmarks and to generate the head coordinate system.

The novelty of our proposal is in the location and estimation of head fiducials landmark using computer vision methods (nasion, periauricular points), which are necessary to find the relative position of electrodes on the 3D head model.

The results of this work constitute a proof of concept for the initial state of a project aimed at the development of an application capable of digitizing the coordinates of electrodes automatically.

## Materials

**FIGURE 2:**
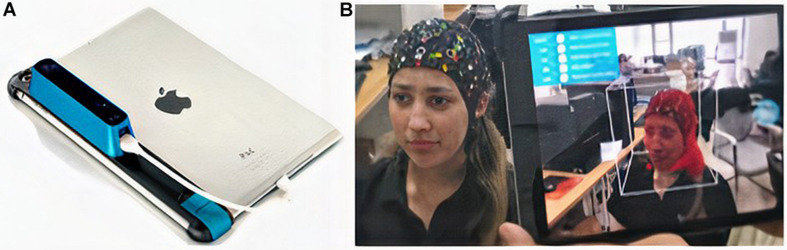
**(A)** ST01 occipital structure sensor (Occipital Inc, 2019) and iPad A1893 (EveryMac.Com, 1996). **(B)** Scanning process around a subject.

For the 3D head model capturing, we propose the model ST01 of the structure sensor manufactured by Occipital Inc., 1801 13th Street Suite 202 Boulder, CO 80302 United States (Figure 2A; Occipital Inc, 2019), which has a precision of 0.5 mm to 40 cm (Occipital Inc, 2018), with an iPad A1893 to scan the subject’s head (Figure 2B), as described on the FieldTrip web page (Homölle and Oostenveld, 2019; Oostenveld, 2019). However, we did not add marks to the fiducial points on the subject’s head. The recommendations are good illuminations and 40 to 60 cm distance between the subject and the scanner. Upon scanning the participant, the data can be delivered by email.

The experimental protocol consisted of scanning the subject’s head with and without an EEG helmet. The experiment was approved by the local Ethical Committee at the University of Electronic Science and Technology of China in compliance with the latest revision of the Declaration of Helsinki for the use of personal images.

The experimental sample was composed of 19 healthy subjects who were recruited as volunteers for this study. All participants gave written informed consent for the publication of any potentially identifiable images included in this article. We also generated a large set of images containing ears for the training of detectors, which could be available on request to the first author.

**FIGURE 3:**
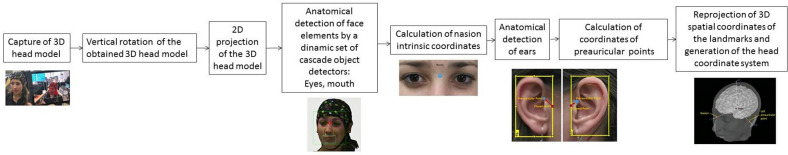
General outline of the proposed method.

Figure 3 shows a general outline of the proposed method.

## Methods

The general procedure is to achieve a 2D projection of each fiducial mark, obtained from its best possible view, to estimate the intrinsic coordinates of each mark, and automatically reproject them to the 3D model of the head to find their spatial coordinates (Homölle and Oostenveld, 2019).

Those operations involve critical elements, as the position of the 3D head model, the detector type to be employed in each situation, the false positive rate of these detectors, the handling of the intrinsic coordinates used for the pixel representation, and the final estimation of fiducials.

**FIGURE 4:**
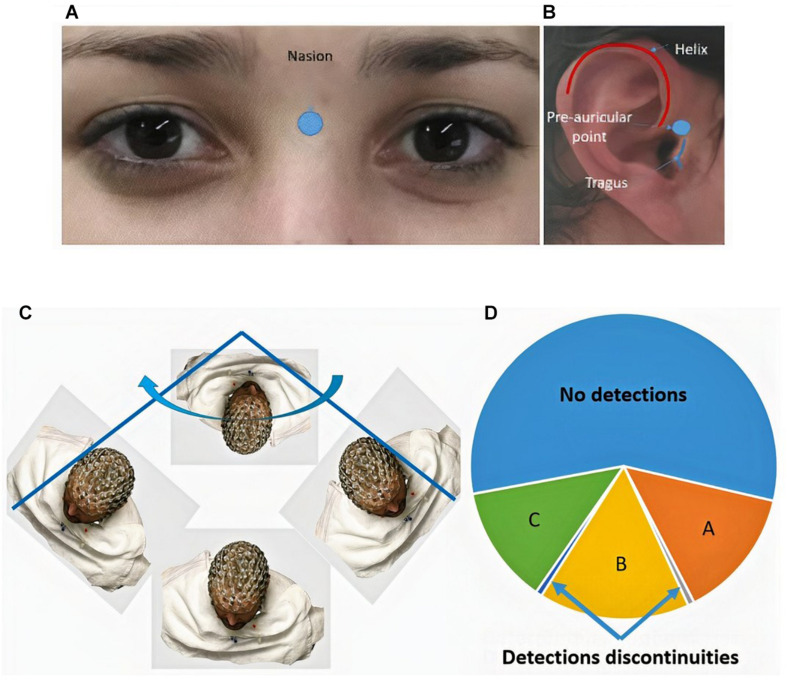
**(A)** Nasion, **(B)** right preauricular point, **(C)** surface rotation for feature detection, and **(D)** detection of continuities and discontinuities.

To manage the obtained images and analytically define the 2D coordinates of the fiducial mark of interest, we consider the following anatomical concepts:

The nasion is the most anterior point of the frontonasal suture that joins the nasal part of the frontal bone and the nasal bones (Collins, 2019), and it marks the midpoint at the intersection of the frontonasal suture with the internasal suture joining the nasal bones. The nasion is visible on the face as a distinctly depressed area directly between the eyes, just superior to the bridge of the nose (Yen, 1960; see Figure 4A).

The preauricular point; is at the posterior root of the zygomatic arch lying immediately in front of the upper end of the tragus (Preauricular Point, 2019; see Figure 4B).

In the next sections, we explain the transversal relationship of these elements with (a) the location of the nasion, (b) the detection of ears and the definition of the preauricular points, and (c) the automatic control of the graphic operations, re-projection, and generation of the coordinate system.

The first mandatory step of our proposal is the vertical rotation of the 3D head model. This step is necessary since the scanner software uses a gyroscope to orient the images considering the gravity direction, which means that the *Z*-axis of the scanned structures is in the direction of gravity and, when shown on the screen, they appear inverted to how they are seen in the real world.

The next step is the 2D projection of the 3D head model. This projection is made for each rotation angle at 360°, achieving 2D frames of the intrinsic coordinate system of pixel representation (Homölle and Oostenveld, 2019; Taberna et al., 2019).

The third step of the proposed method consists in the automatic detection on the obtained 2D projection of face elements (face, eyes, nose, mouth, and ears) that are necessary to achieve the anatomical references that later allow defining the coordinates of the element known as nasion and preauricular points, as a point, that are, the coordinates (x, y) of the pixel where these fiducials are located.

### The Location of the Nasion

The process of nasion location comprises two main steps: (a) the detection of anatomical face elements (face, mouth, nose, and eyes) and (b) the estimation of intrinsic coordinates of the nasion.

#### Detection of Anatomical Face Elements

To carry out this task, we propose a set of detectors described in the computer vision literature. For face detection in the frontal position, we adopt the use of the detector described in The MathWorks Inc (2017a); for detection of eyes, mouth, and nose, we propose the use of the other three detectors (Freund and Schapire, 1997; Castrillón et al., 2007; Lienhart et al., 2007; Acosta Solórzano and Cuellar Ramírez, 2016).

The face detector is capable of detecting faces that are upright and forward, allowing modeling higher-order dependencies between facial features. The eye detector can find eyes, right and left separately; in the same way with the other two is possible to detect the nose and mouth, locking them in different boxes.

The main characteristic of these detectors is that they were trained through the classic algorithm of Viola and Jones (Viola and Jones, 2001; McCane and Novins, 2003; Castrillón et al., 2007; Lienhart et al., 2007; Castrillón et al., 2011). The image representation called an integral image, allows a high-speed computation of the features used by the detectors. The learning algorithm based on Adaboost will enable us to select a small number of features from the initial set and to obtain a cascade of simple classifiers to discriminate them. The classifiers were trained to detect the region of each anatomical element (face, nose, mouth, eyes). This region includes the bounding box that encompasses each anatomical elements.

The detection process can produce a set of face images in which all anatomical elements were detected, another group in which only some anatomical elements were detected, and another set in which any single anatomical elements were detected (Figure 4C). These different sets arise due to the effect of rotation of the 3D head model in the face pose.

In this work, we propose to take the face image that presents the best frontal pose. To obtain this image, we offer an algorithm that allows us to define which arc has been the one that has no discontinuities in the detection and therefore has better orientation of the frontal face pose (section B in Figure 4D).

**FIGURE 5:**
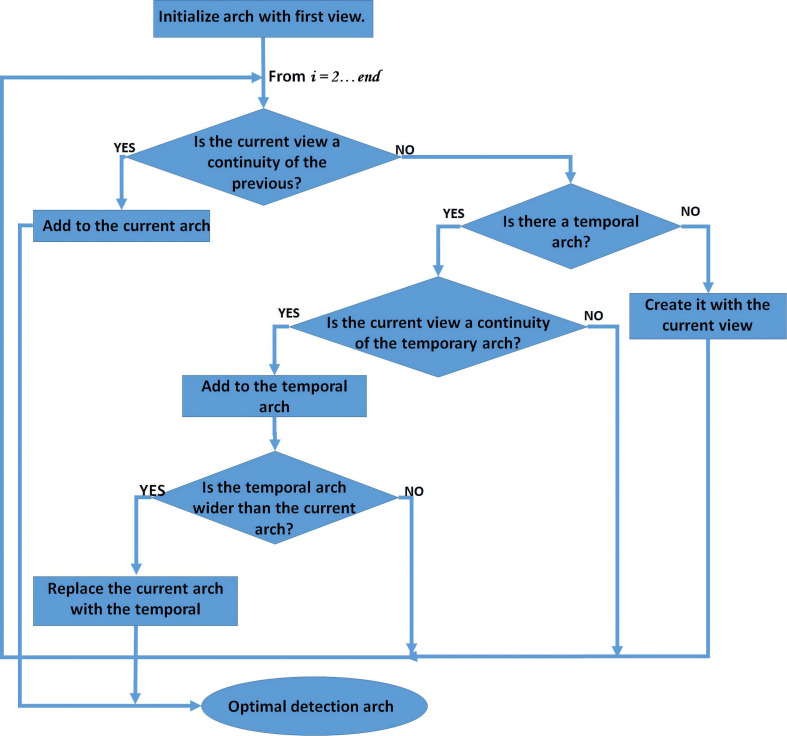
General outline of the proposed algorithm to find the optimum detection arc.

Figure 5 shows the proposed algorithm for optimal arc detection, to be applied after all the rotation angles with successful detection (but with discontinuities) were obtained.

This algorithm is a critical point in the proposed method. It is necessary to achieve 2D projections where the fiducial anatomical points are visualized. Its success depends mainly on the quality of the process of scanning the subject’s head since the occurrence of discontinuities in the detection of face anatomical elements can cause the selection of the wrong arc and the subsequent occurrence of false positives in the detection of ears.

#### Estimation of Intrinsic Coordinates of the Nasion

**FIGURE 6:**
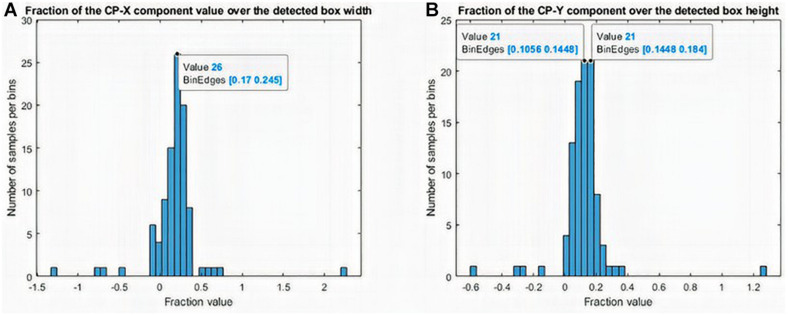
Histogram of the behavior of the distance between the preauricular point and the closest point on the right ear. **(A)** *x-*component variation and **(B)** *y*-component variation.

To obtain the nasion intrinsic coordinates over the selected face image, we propose the equation, (1), which is based on the geometrical structure of the bounding box returned by the detectors (see Figure 6), taking in to account the fact that we already have the bounding boxes for both eyes (right eye [y1, x1, w1, h1]; left eye [y2, x2, w2, h2]) and the nose [y3, x3, w3, h3]. Accordingly, the nasion intrinsic coordinates are

(1) $$\left[x,y\right]=\left\{\left[\frac{x\,2+x\,1}{2}+\frac{h\,2+h\,1}{4}\right],\left[\frac{y\,1+y\,2+w\,2}{2}+y\,3+\frac{w\,3}{2}\right]\right\}$$

The robustness of the facial detection process is achieved by rotating the detectors around the 3D model in a 1-degree angular interval. Only in cases where the scanner was inaccurate, the detection of capturing the facial image fails.

### Ear Detection and Preauricular Points Definition

We empirically verified that the best lateral projection of the subject’s faces to completely see the ears is obtained by rotating the 3D model ± 87° from the angle in which the best frontal view was defined. The correct selection of this lateral view is essential for the best performance of ear detectors.

#### Ear Detection

**FIGURE 7:**
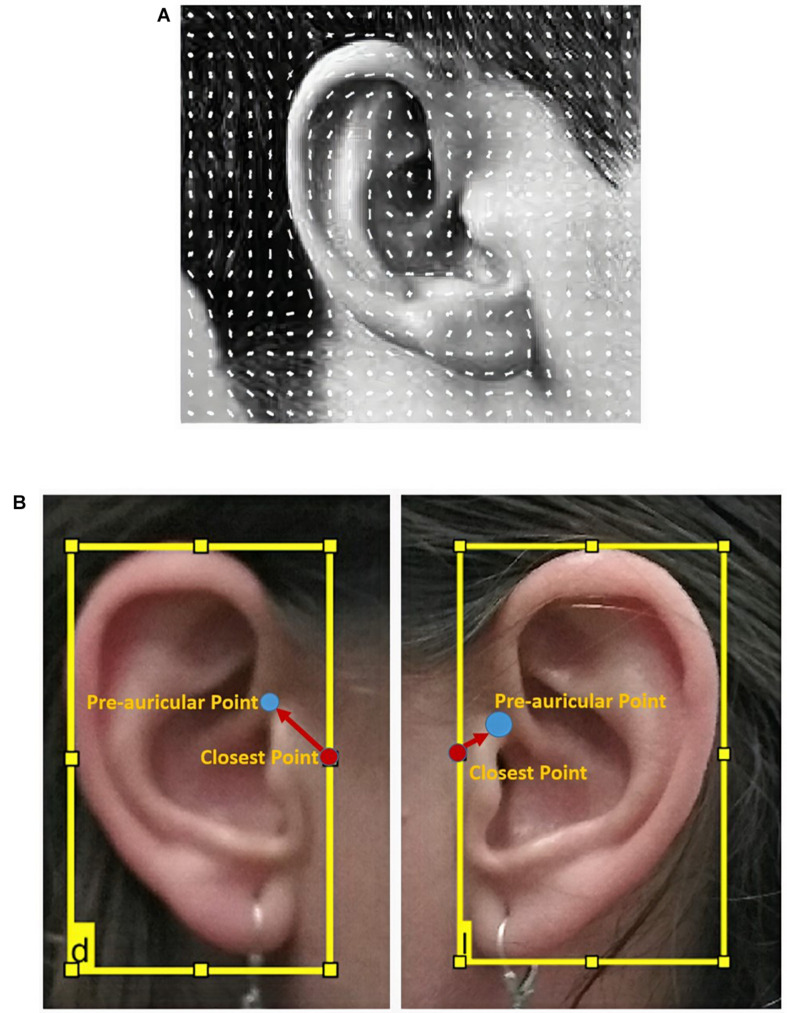
**(A)** Typical histogram of oriented gradients of one ear and **(B)** correction of the preauricular points.

For this work, we propose the use of an ear detector trained through the classic algorithm of Viola and Jones (Hicks and Steffler, 1995; Viola and Jones, 2001; McCane and Novins, 2003). The specific technique chosen to train the detectors was HOG. This technique is based on the number of occurrences of gradient orientations in localized portions of an image (McCane and Novins, 2003). The HOG is computed on a dense grid of uniformity spaced cells and uses overlapping local contrast normalization for improved accuracy, thereby achieving the feature pattern of the ears, as shown in Figure 7A.

The implemented training method and the corresponding code are available and shared on GitHub (Gallego, 2019).

The detector stages were designed to reject negative samples as fast as possible. The assumption is that the vast majority of windows do not contain the object of interest. However, true positives are rare but necessary, and thus, it is worth taking the time to verify true positives. The definitions for these terms are

• A real positive occurs when a positive sample is correctly classified.
• A false positive occurs when a negative sample is erroneously classified as positive.
• A false negative occurs when a positive sample is erroneously classified as negative.

For this method to work effectively, each stage in the cascade must have a low false-negative rate. If a step incorrectly labels an object as negative, the classification stops, and the mistake cannot be corrected. However, each step can have a high false alarm rate (FAR). Even if the detector incorrectly labels a non-object as positive, the mistake can be corrected in subsequent stages.

The overall FAR of the cascade classifier is *f*^*s*^, where *f* is the FAR per stage in the range (0, 1), and *s* is the number of steps. Similarly, the overall correct positive rate is *t*^*s*^, where *t* is the actual positive rate per stage in the range (0, 1). Thus, adding more steps reduces the overall FAR but also reduces the overall correct positive rate (The MathWorks Inc, 2017b).

#### Preauricular Points Definition

Once the ears were detected, by applying specific detectors to a projection of the 3D model rotated ± 87° (from the angle corresponding to the best view where the nasion was found), the intrinsic coordinates of the closest point (*CP*) to the preauricular point are calculated by equations (2) and (3) for the right and the left ear, respectively (corresponding to the red point in Figure 7B).

(2) $$R\,i\,g\,h\,t\,\_\,C\,P\,\left(x,y\right)=\left\{\left[X_{l\,e\,f\,t}+w\,i\,d\,t\,h\right],\left[Y_{t\,o\,p}+\frac{h\,e\,i\,g\,h\,t}{2}\right]\right\}$$

(3) $$L\,e\,f\,t_{P\,P}\,\_\,C\,P\,\left(x,y\right)=\left\{\left[X_{l\,e\,f\,t}\right],\left[Y_{t\,o\,p}+\frac{h\,e\,i\,g\,h\,t}{2}\right]\right\}$$

(*X*_*left*_,*Y*_*top*_) refers to the upper left corner of the bounding boxes that enclose the ears, while width and height are the width and height measurements, respectively, of the preauricular point according to the closest point (see Figure 6B).

To measure the accuracy of the described close point (*CP*) detection process, we calculated the distance between the preauricular point and the closest point on the right ear. Figure 6 shows the *x-*component variation (6a) and *y*-component variation (6b) in a set of 100 different pairs of right and left ears.

To minimize the errors with respect to the real anatomical preauricular point shown in Figure 6B, we propose a correction according to equations (4) and (5) as a function of the box structure dimensions. The constants in these equations minimize the sum of absolute values of the errors. They were determined employing an exhaustive grid search.

(4) $$R\,i\,g\,h\,t\,\_\,P\,P\,\left(x,y\right)=\left\{\begin{array}{c} \left[X_{l\,e\,f\,t}+\frac{8}{10}*w\,i\,d\,t\,h\right],\\ \left[Y_{t\,o\,p}+0.36*h\,e\,i\,g\,h\,t\right] \end{array}\right\}$$

(5) $$L\,e\,f\,t\,\_\,P\,P\,\left(x,y\right)=\left\{\begin{array}{c} \left[X_{l\,e\,f\,t}+\frac{w\,i\,d\,t\,h}{5}\right],\\ \left[Y_{t\,o\,p}+0.36*h\,e\,i\,g\,h\,t\right] \end{array}\right\}$$

This correction represents the variation of components *x* and *y* of the intrinsic coordinates when moving from the *CP* to the visually estimated preauricular point. The detected right ear box width is shrunken by 20% and its height by 14% *y*-axis of the *x* and *y* CP coordinate values, respectively. The detected left ear box width is increased by 20%, and its height decreased by 14% of the *x* and *y* CP coordinate values, respectively.

This correction can be performed because of the anatomical shape of the ears and the ROI defined in the Image Labeler application during the training process, in which 90% of the time, this transformation was valid (Figure 7B).

Because this is an estimation, an error will be incurred, but the estimated coordinates will always be more accurate than the *CP*. Also, fiducial landmarks are not related to a specific coordinate because they are anatomical points that can be represented with several intrinsic coordinates corresponding to 1 pixel.

The success of the aforementioned operations, toward the intrinsic coordinates of the preauricular points, will be affected only by the occurrence of a false positive or a false negative by ear detection. This probability will be analyzed while describing the process of designing the detectors in “Results and Discussion” section.

Nevertheless, the robustness of this procedure is derived from the dynamic use of several ear detectors with different FARs and the recursive use of the combined set of possible detectors over a 10-degree arch of the projected view, as will be mentioned later.

### Automatic Control of Graphics Operations, Re-projection, and Coordinate System Generation

At this point, we already have the fiducial points, but only a single (*x*,*y*) point of an intrinsic coordinate system; these data pertain to a projection in two dimensions of the subject’s head surface.

However, to conduct an automatic re-projection, it is necessary to guarantee certain conditions, such as

• To know where exactly on the screen is the model that contains the surface of the subject’s head.
• To guarantee that this model inherits the dimensions of the images with the fiducial’s projections in 2D when they have correct size resolution, that is when the pixels in the images have the same size as the pixels on the screen.
• To have full control over mouse movement on-screen and click event. Before initiating any click event, we need to ensure that the graphical object overlies all other windows.

**FIGURE 8:**
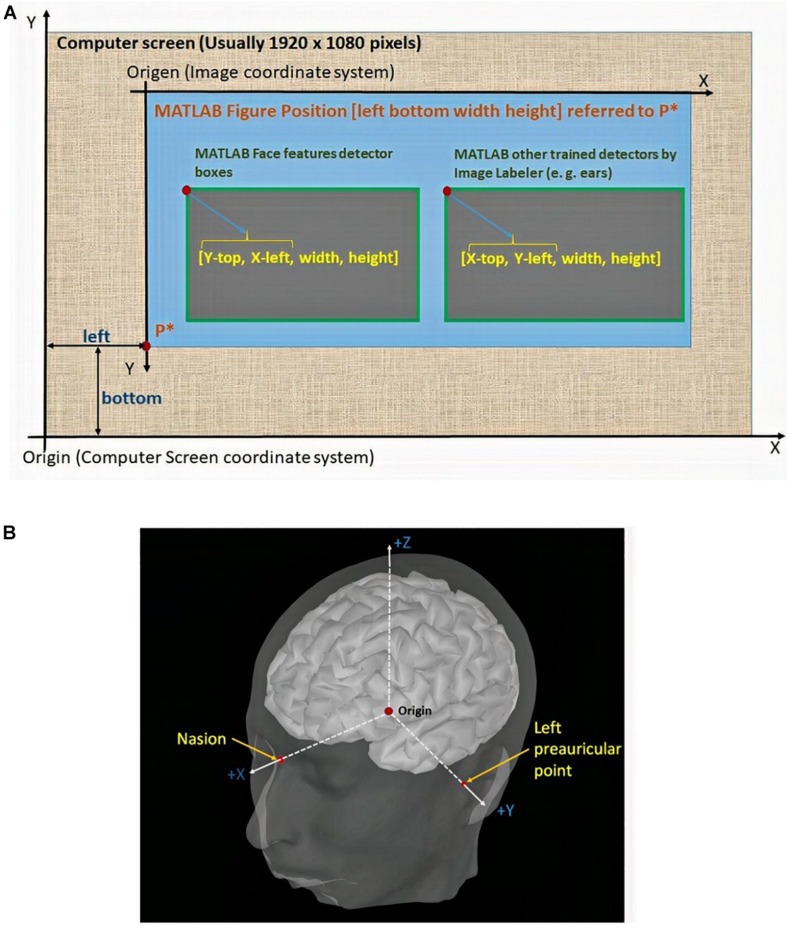
**(A)** Differences among the intrinsic coordinate system of the pixels on the screen, the MATLAB figure positions, and the box structures returned by the detectors. **(B)** Subject coordinate system (ShistCS/CTF).

To fulfill the first and third conditions, we consider the relationship between the coordinate systems shown in Figure 8A.

The new mouse position is mapped to the screen coordinate system, as a function of the intrinsic coordinates of the fiducial points in two dimensions. Thus, the axes are positioned inside the figure, which is itself inside the screen. These transformations are expressed as follows:

Let the fiducial intrinsic coordinate be*F* = [*F*_*x*_,*F*_*y*_].

The figure position is as follows:

$$F\,i\,g=\left[F\,i\,g_{l\,e\,f\,t},F\,i\,g_{b\,o\,t\,t\,o\,m},F\,i\,g_{w\,i\,d\,t\,h},F\,i\,g_{h\,e\,i\,g\,h\,t}\right].$$

Where the position of the *x*-axis is as follows:

$$A\,x=\left[A\,x_{l\,e\,f\,t},A\,x_{b\,o\,t\,t\,o\,m},A\,x_{w\,i\,d\,t\,h},A\,x_{h\,e\,i\,g\,h\,t}\right].$$

The number of rows for the intrinsic coordinate system in the image is *A*.

Then, the coordinates sent to the mouse pointer will be:

$$\left(x,y\right)=[F_{y}+Fig_{l\,e\,f\,t}+Ax_{l\,e\,f\,t},A-F_{y}+Fig_{b\,o\,t\,t\,o\,m}+Ax_{b\,o\,t\,t\,o\,m}$$

Once the click event is captured, the next step is to systematically find the (*x*,*y*,*z*) points selected over the surface. This task can be done with the FieldTrip Toolbox (8) private function *select3D*, which is based on a projection transformation and the 2D crossing test (the Jordan curve theorem; Jordan, 1894).

Finally, having the (*x*,*y*,*z*) coordinates corresponding to the nasion and both preauricular points, we generate the subject coordinate system (SCS/CTF) using the FieldTrip Toolbox, which is based on the fiducial locations, as shown in Figure 8B.

## Results and Discussion

For the validation of the proposed method, we implemented its pipeline on MATLAB R2018b. We used the Image Processing and Computer Vision Toolbox of MATLAB for facial feature recognition and object detector, and the FieldTrip Toolbox for some additional operations.

For the tasks of detecting the face, eyes, and mouth, we use the detectors implemented in MATLAB. Detectors based on the classification models “FrontalFaceCART,” “LeftEye,” “RightEye,” “Mouth,” and “Nose” (Freund and Schapire, 1997; Castrillón et al., 2007; Acosta Solórzano and Cuellar Ramírez, 2016; The MathWorks Inc, 2017a).

The experimental design was aimed to measure the accuracy of our proposal in the detection of facial features using the MATLAB detectors for the definition of the nasion, the detection of the ears for defining the preauricular points (both in two dimensions), the re-projection process, and the generation of the coordinate system.

During the experiments, the 3D models of the head of 19 volunteers were captured using the previously described sensor.

The output produced by the scanning process is a compressed file containing three files, all of them with the name “Model” but having.*obj*,.*jpg*, and.*mtl* extensions. These files were extracted into the current MATLAB direction and employed for further image processing.

### Experimental Results for Nasion Location

**FIGURE 9:**
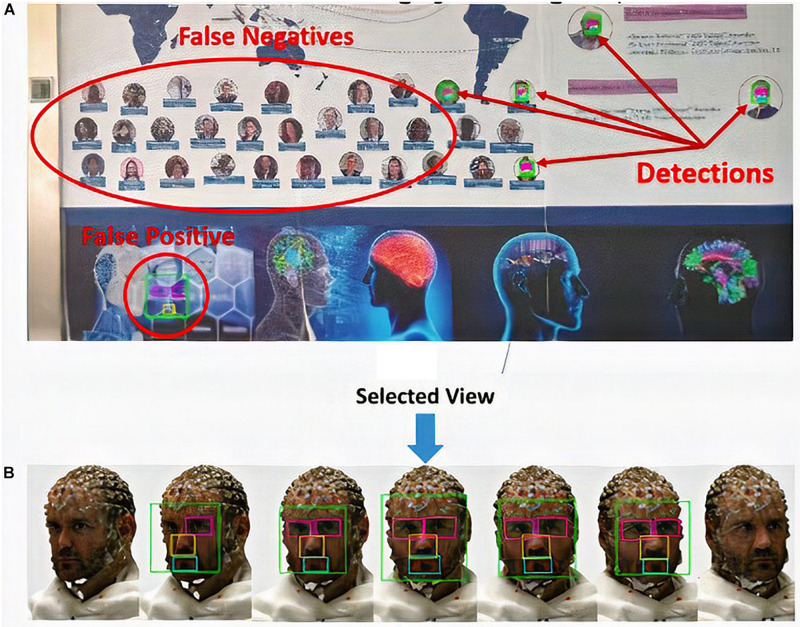
Facial feature detection: **(A)** false-positive and false-negative result for the detection of facial features and **(B)** selection of the best view.

For evaluation of detector accuracy, we used the probability of not detecting an object (false negative) or detecting an object for which it has not been trained (false positive), as shown in Figure 9A.

In the search procedure for facial features on the surface projection, the number of detected views depends only on the chosen rotation angle (turned in step-by-step intervals) and the conditions of the facial features on the image pixels (Figure 9B).

Table 1 shows the results obtained in measuring the effectiveness of the detectors.

**TABLE 1:** Facial feature detection efficacy as a function of the rotation angle referenced to the frontal view.

Subject number	Arch (°)
1	137 < α < 196
2	179 < α < 242
3	202 < α < 235
4	178 < α < 221
5	168 < α < 220
6	150 < α < 207
7	193 < α < 234
8	147 < α < 204
9	160 < α < 211
10	156 < α < 210
11	151 < α < 196
12	153 < α < 226
13	152 < α < 204
14	143 < α < 206
15	158 < α < 209
16	192 < α < 195
17	164 < α < 204
18	162 < α < 210
19	146 < α < 217

The facial elements were detected in 100% of the models in the frontal position with a minimum rotation range of 3°. In Table 1, the column “Arc (°)” represents the angle ranges in which the detection was successful.

**FIGURE 10:**
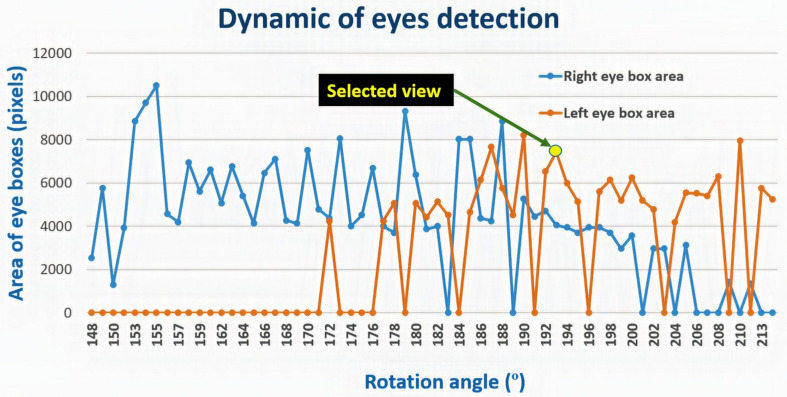
Dynamics of the detection of the eyes for subject 16 (listed in Table 2) showing the eye box area vs. the rotation angle.

Each model has a minimum arc of rotation in which the facial elements are detected, but this does not mean that the detectors are not able to find them at other intervals. Figure 10 shows the dynamic results of eye detection for subject number 16 in Table 1, which represents the worst case among the models with respect to the selection of the best view for detection. The angles in the intervals of 148–171° and 206–208° with zero value indicate that the left eye was not detected in those intervals; these angular ranges correspond to model views where the left eye is not visible.

In this case, the view corresponding to rotation angle 193° contains the eye detections (bounding box) with the highest number of pixels, then the arc to which it belongs (192 < α < 195) is defined as the minimum for successful detection. This decision criterion is because the eyes detected in a view that is not completely frontal always occupy a smaller area in the image than in the more frontal views, as shown in Figure 9B.

To ensure that facial elements are detected in a frontal view, we set the search range between 0° and 360°. Once we have the view projections of the model at each angle, we choose the most symmetric view according to the spatial distribution of bounding boxes of the detected elements. This procedure enables better precision in the subsequent location of the nasion. An example of this process is shown in Figure 9B, which shows the sequence of images in which facial elements were detected and the image selected under this criterion.

In general, the success of the detection of facial elements also depends on several elements such as the pose of the face, the presence of glasses, and the occlusion of the facial features by the hair and the facial expression of the person or other anatomical deformations. To avoid false positives, we recommend that people at the time of model capture do not wear plaid blouses or shirts, as this could generate pixelated images during the scanning process.

**FIGURE 11:**
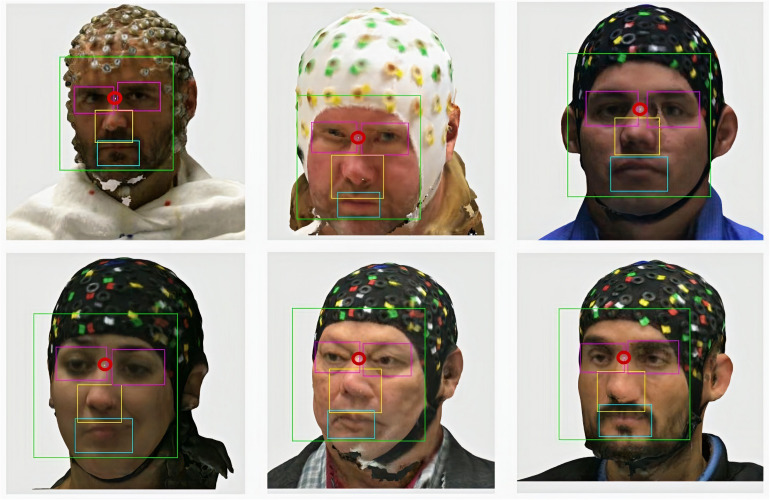
Facial feature detection and nasion location results.

Figure 11 shows some examples of the detection of facial elements and the calculation of the nasion coordinates according to equation (1), considering the symmetry criterion of the bounding boxes (in pixels) detected for the eyes and nose. To represent the nasion coordinates, we place a blank pixel in the image highlighted by a red circle.

### Experimental Results for Detection of the Ears and Definition of Preauricular Points

The ear detectors were manually built (as mentioned in “Methods” section) using two sessions on the Image Labeler app in the Computer Vision Toolbox with 2,308 positive images for the right ear, 2,300 positive images for the left ear, and 1,323 negative samples for both detectors.

To perform the detector training, two intervals of FAR values were taken, one from 0.3 to 0.8 with a step of 0.1 and the other from 0.8 to 0.9 with step of 0.05 (second column of Table 2).

**TABLE 2:** Design of the ear detectors.

Type of detector	FAR per stage	Number of stages resulting	Total FAR
Left ear	0.3	11	0.000002
	0.4	14	0.000003
	0.5	17	0.000008
	0.6	19	0.000061
	0.7	25	0.000134
	0.8	33	0.000634
	0.85	35	0.003386
	0.9	41	0.013303
Right ear	0.3	8	0.000066
	0.4	12	0.000017
	0.5	14	0.000061
	0.6	15	0.000470
	0.7	19	0.001139
	0.8	21	0.009223
	0.85	24	0.020233
	0.9	25	0.071789

The results obtained in the training process are shown in Table 2. The third and fourth columns of the table represent the resulting number of stages for each set FAR value and the total FAR value obtained in the training process, respectively.

Considering these results, we propose to use all detectors dynamically, starting with the lowest FAR (represented in bold in Table 2) and switching to the next detector when no ear is detected, and to repeat this procedure recursively for a set of views corresponding to a range of 10°. This procedure will guarantee that no false positives are obtained and that a single true positive is acquired.

**FIGURE 12:**
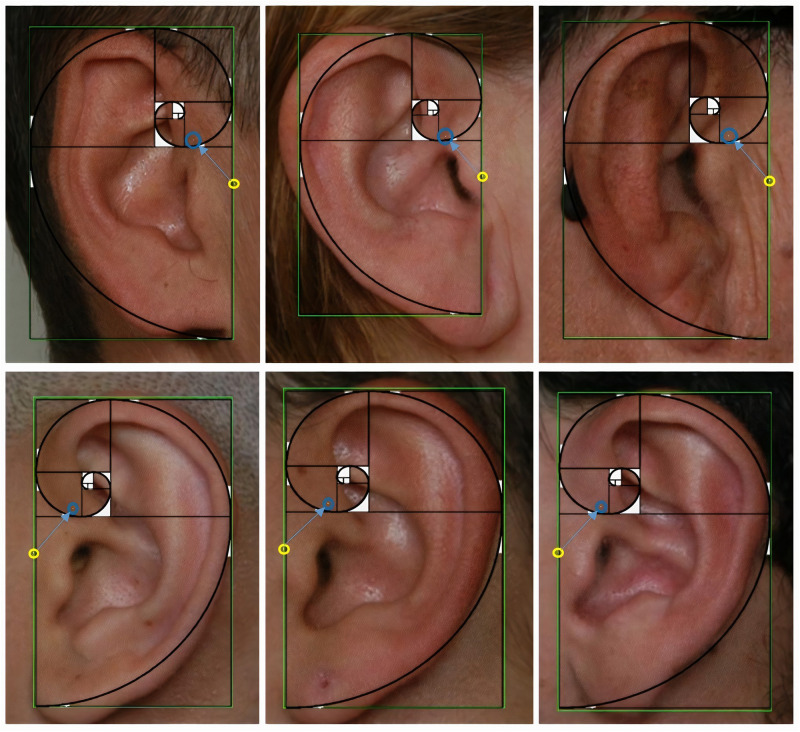
Ear detection results, correction of the closest point toward the preauricular point, and verification of the coordinates with the Fibonacci spiral.

Figure 12 shows the results obtained for the detection of the right and left ear, as well as the CP, represented as a yellow circle. It also the correction toward the right preauricular point in a blue circle according to Eqs (4) and (5).

Another choice for correcting the preauricular point is to fit the detected box to a Fibonacci spiral by drawing at least four sections of the spirals and marking the fourth tangent point of the spiral.

This procedure has an additional meaning because the learning process carried out by the detectors based on oriented gradient histograms allows not only to detect the invariant image characteristics but also to frame them in a constant proportion. It was further employed to visually verify the obtained coordinates after applying Eqs (4) and (5) and using the φ proportion four consecutive times.

### Experimental Results for Reprojection Process and Generation of the Subject Coordinate System

To reproject 2D points onto a 3D surface corresponding to the nasion and preauricular points, we implemented the procedure based on the head model and the Jordan curve theorem with the FieldTrip Toolbox functions (as described in “Methods” section).

**FIGURE 13:**
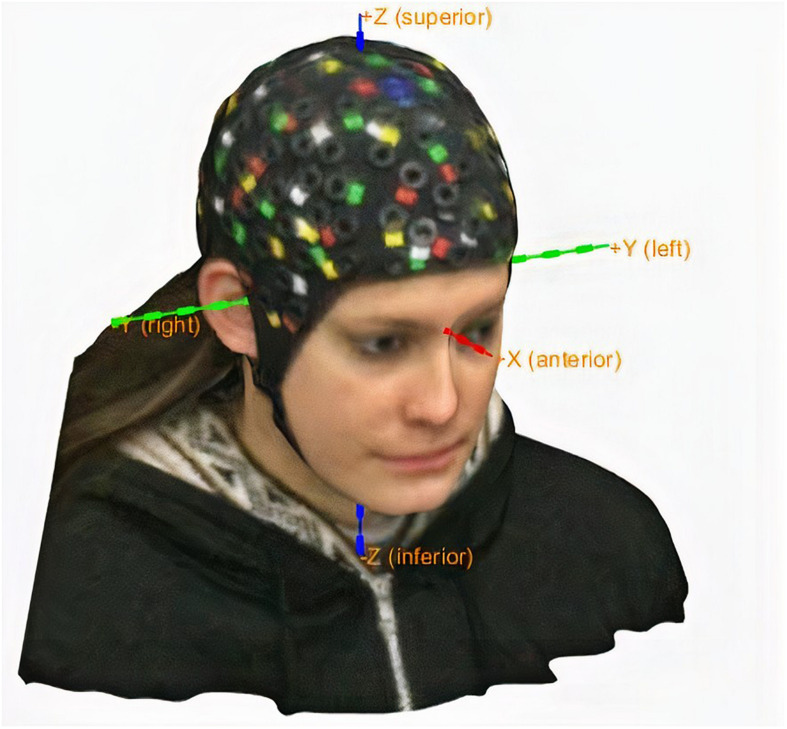
Subject coordinate system.

Figure 13 shows the final generated SCS employing the entire automatic fiducial detection described process.

Concerning the processing time required to define the SCS (starting with the reading of the obj file), both the manual procedure and the automatic procedure took an average of 2 min per subject on a Core i7 personal computer with 16 gigabytes of RAM.

The routines that consumed more time were those that read the object file and the exploratory procedure to detect facial features toward the nasion coordinates.

The error incurred in this procedure is attributable to the precision of the manual procedure because an anatomical point can be selected with multiple points in an image belonging to an intrinsic coordinate system.

## Conclusion

The location of fiducial points has long been a problem when co-registering EEG sensors with MRI results. The proposed method has the advantages of being a cheap and fast alternative to obtain a reference system for spatial coordinates; furthermore, the proposed approach will allow the coordinates of EEG sensors to be defined based on real locations over a subject’s head and not on templates.

Detectors based on cascade classifiers offer a fast alternative to detect facial features and allow to define fiducial landmarks in two dimensions. The only limitation of this automatic fiducial location procedure is related to the 3D model capture. It must be performed under good illumination conditions; moreover, the scanner must be moved slowly around the subject’s head to acquire all the facial features while simultaneously guaranteeing that no artifacts will hide the facial characteristics, that the detectors will do their job, and that the subject’s coordinates system (SCS) will be generated successfully.

The robustness of the proposed method is in the exploratory procedure to detect the facial features; in the case of the ears, it is in the dynamic use of the detectors, beginning with those having a relatively low false alarm rate (FAR) and then switching to those with a higher FAR when the ears were not detected. Besides, all detectors were used recursively as a function of the projection angle.

Because this study constitutes a proof of concept, the final SCS will allow the coordinates of the EEG electrodes to be in a homogeneous reference coordinate system, which will be explored in future work.

The same paradigm could be used to project a 3D object into 2D images with an intrinsic coordinate system to easily find electrodes and reproject their coordinates into three dimensions, thereby generating an electrode coordinate cloud.

## Data Availability Statement

All datasets generated for this study are freely available in https://drive.google.com/drive/folders/13cx01b3OQFp68StVMc6dCHak0T-c6Cr6.

## Ethics Statement

The studies involving human participants were reviewed and approved by the University of Electronic Sciences and Technology of China. The patients/participants provided their written informed consent to participate in this study. Written informed consent was obtained from the individual(s) for the publication of any potentially identifiable images or data included in this article.

## Author Contributions

The contribution of EGM to the presented results were the design of the research, the preparation of the materials and the development of the software. AG contributed with the mathematical approach to validate the experimental procedure, the recruitment and collection of the data, and the final processing of the results. PV-S conceived the idea of the research and with MB-V and EG-L supervised the process and did the final revision. EGM and AGM contributed equally to this research. All authors contributed to the article and approved the submitted version.

## Conflict of Interest

The authors declare that the research was conducted in the absence of any commercial or financial relationships that could be construed as a potential conflict of interest.

## References

[B1] Acosta Solórzano W. F. Cuellar Ramírez H. (2016). *Detección y Reconocimiento de Rostros Usand**o MATLAB.* Available online at: http://repositorio.utp.edu.pe/handle/UTP/541 (accessed June, 2019).

[B2] Binnie C. D. Dekker E. Smit A. Van Der Linden G. (1982). Practical considerations in the positioning of EEG electrodes. *Electroencephalogr. Clin. Neurophysiol.* 53 453–458. 10.1016/0013-4694(82)90010-4 6175507

[B3] Castrillón M. Déniz O. Guerra C. Hernández M. (2007). ENCARA2: Real-time detection of multiple faces at different resolutions in video streams. *J. Vis. Commun. Image Represent.* 18 130–140. 10.1016/j.jvcir.2006.11.004

[B4] Castrillón M. Déniz O. Hernández D. Lorenzo J. (2011). A comparison of face and facial feature detectors based on the Viola–Jones general object detection framework. *Mach. Vis. Appl.* 22 481–494.

[B5] Chatrian G. E. Lettich E. Nelson P. L. (1985). Ten percent electrode system for topographic studies of Spontaneous and Evoked EEG activities. *Am. J. EEG Technol.* 25 83–92. 10.1080/00029238.1985.11080163

[B6] Collins (2019). *Definition of “Nasion”.* Available online at: https://www.collinsdictionary.com/dictionary/english/nasion (accessed June, 2019).

[B7] Dalal S. S. Rampp S. Willomitzer F. Ettl S. (2014). Consequences of EEG electrode position error on ultimate beamformer source reconstruction performance. *Front. Neurosci.* 8:42. 10.3389/fnins.2014.00042 24653671 PMC3949288

[B8] De Munck J. C. Vijn P. C. M. Spekreijse H. (1991). A practical method for determining electrode positions on the head. *Electroencephalogr. Clin. Neurophysiol.* 78 85–87. 10.1016/0013-4694(91)90023-w 1701720

[B9] Delorme A. Makeig S. (2004). EEGLAB: an open source toolbox for analysis of single-trial EEG dynamics including independent component analysis [Internet]. *J. Neurosci. Methods* 134 9–21. 10.1016/j.jneumeth.2003.10.009 15102499

[B10] Engels L. De Tiège X. Beeck M. Warzée N. (2010). Factors influencing the spatial precision of electromagnetic tracking systems used for MEG/EEG source imaging. *Neurophysiol. Clin.* 40 19–25. 10.1016/j.neucli.2010.01.002 20230932

[B11] EveryMac.Com (1996). *Mac & Apple Devices–EveryMac.com’s Ultimate Mac Lookup.* Available online at: https://everymac.com/ultimate-mac-lookup/?search_keywords=A1893 (accessed June, 2019).

[B12] Freund Y. Schapire R. E. (1997). A Decision-theoretic generalization of on-line learning and an application to boosting. *J. Comput. Syst. Sci.* 55 119–139. 10.1006/jcss.1997.1504

[B13] Gallego E. E. (2019). *Matlab Code for Automatic Fiducial Detection with Computer Vision”.* Available online at: https://github.com/elieserernesto/Fiducial_Detection (accessed June, 2019).

[B14] Hicks F. E. Steffler P. M. (1995). Comparison of finite element methods for the St. Venant equations. *Int. J. Numer. Methods Fluids* 20 99–113. 10.1002/fld.1650200202

[B15] Homölle S. Oostenveld R. (2019). Using a structured-light 3D scanner to improve EEG source modeling with more accurate electrode positions. *J. Neurosci. Methods* 326:108378. 10.1016/j.jneumeth.2019.108378 31376413

[B16] Jordan C. (1894). Cours d’analyse de l’ecole polytechnique. *Bull. Amer. Math. Soc.* 3 135–141.

[B17] Klem J. H. (1961). The ten twenty electrode system: international federation of societies for electroencephalography and clinical neurophysiology. *Am. J. EEG Technol.* 1 13–19. 10.1080/00029238.1961.11080571

[B18] Koessler L. Cecchin T. Ternisien E. Maillard L. (2010). 3D handheld laser scanner based approach for automatic identification and localization of EEG sensors. *Conf. Proc. IEEE Eng. Med. Biol. Soc.* 2010 3707–3710. 10.1109/IEMBS.2010.5627659 21097050

[B19] Koessler L. Maillard L. Benhadid A. Vignal J.-P. Braun M. Vespignani H. (2007). Spatial localization of EEG electrodes. *Neurophysiol. Clin.* 37 97–102. 10.1016/j.neucli.2007.03.002 17540292

[B20] Lienhart R. Kuranov A. Pisarevsky V. (2007). *Empirical Analysis of Detection Cascades of Boosted Classifiers for Rapid Object. J Vis Commun Image Represent.* Available online at: https://link.springer.com/chapter/10.1007/978-3-540-45243-0_39 (accessed June, 2019).

[B21] McCane B. Novins K. (2003). *On Training Cascade Face Detectors Brendan. Image Vis Comput NZ, Image Vis Comput NZ*. Available online at: http://www.eng.auburn.edu/~troppel/internal/sparc/TourBot/TourBotReferences/Haar/IVCNZ_43.pdf (accessed June, 2019).

[B22] Nelson C. V. Jacobs B. C. (2004). *Magnetic Sensor System for Fast-Response, High Resolution, High Accuracy, Three-Dimensional Position Measurements*. Google Patents. US US9757605B2.

[B23] Occipital Inc (2018). *What are the Structure Sensor’s Technical Specifications?.* Available online at: https://support.structure.io/article/157-what-are-the-structure-sensors-technical-specifications (accessed June, 2019).

[B24] Occipital Inc (2019). *Structure Sensor, 3D Scanning. Augmented Reality. Instant Measurements.* Available online at: https://store.structure.io/buy/structure-sensor (accessed June, 2019).

[B25] Oostenveld R. (2019). *Localizing Electrodes Using a 3D-Scanner.* Available online at: http://www.fieldtriptoolbox.org/tutorial/electrode/ (accessed June, 2019).

[B26] Oostenveld R. Fries P. Maris E. Schoffelen J. M. (2011). FieldTrip: Open source software for advanced analysis of MEG, EEG, and invasive electrophysiological data. *Comput. Intell. Neurosci.* 2011:156869. 10.1155/2011/156869 PMC3021840 21253357

[B27] Oostenveld R. Praamstra P. (2001). The five percent electrode system for high-resolution EEG and ERP measurements. *Clin. Neurophysiol.* 112 713–719. 10.1016/s1388-2457(00)00527-7 11275545

[B28] Preauricular Point (2019). *Definition of Preauricular Point by Medical Dictionary [Internet].* Available online at: https://medical-dictionary.thefreedictionary.com/preauricular+point (accessed June, 2019).

[B29] Taberna G. A. Marino M. Ganzetti M. Mantini D. (2019). Spatial localization of EEG electrodes using 3D scanning. *J. Neural Eng.* 16:026020. 10.1088/1741-2552/aafdd1 30634182

[B30] The MathWorks Inc (2017a). *Detect Objects Using the Viola-Jones Algorithm.* Available online at: https://www.mathworks.com/help/vision/ref/vision.cascadeobjectdetector-system-object.html (accessed June, 2019).

[B31] The MathWorks Inc (2017b). *Train a Cascade Object Detector.* Available online at: https://la.mathworks.com/help/vision/ug/train-a-cascade-object-detector.html (accessed June, 2019).

[B32] Viola P. Jones M. (2001). “Rapid object detection using a boosted cascade of simple features,” in *Proceedings of the 2001 IEEE Computer Society Conference on Computer Vision and Pattern Recognition CVPR 2001*, Kauai, HI, I–511–I–518.

[B33] Yen P. K. J. (1960). Identification Of landmarks in cephalometric radiographs. *Angle Orthod.* 30 35–41.

